# A New Method for Optimizing Sepsis Therapy by Nivolumab and Meropenem Combination: Importance of Early Intervention and CTL Reinvigoration Rate as a Response Marker

**DOI:** 10.3389/fimmu.2021.616881

**Published:** 2021-03-01

**Authors:** Avi Gillis, Anat Ben Yaacov, Zvia Agur

**Affiliations:** Institute for Medical Biomathematics (IMBM), Bene Ataroth, Israel

**Keywords:** inflammation, mathematical model, intensive care, immunosuppression, PD-1, fitness landscape, simulation, nivolumab

## Abstract

**Background:** Recently, there has been a growing interest in applying immune checkpoint blockers (ICBs), so far used to treat cancer, to patients with bacterial sepsis. We aimed to develop a method for predicting the personal benefit of potential treatments for sepsis, and to apply it to therapy by meropenem, an antibiotic drug, and nivolumab, a programmed cell death-1 (PD-1) pathway inhibitor.

**Methods:** We defined an optimization problem as a concise framework of treatment aims and formulated a fitness function for grading sepsis treatments according to their success in accomplishing the pre-defined aims. We developed a mathematical model for the interactions between the pathogen, the cellular immune system and the drugs, whose simulations under diverse combined meropenem and nivolumab schedules, and calculation of the fitness function for each schedule served to plot the fitness landscapes for each set of treatments and personal patient parameters.

**Results:** Results show that treatment by meropenem and nivolumab has maximum benefit if the interval between the onset of the two drugs does not exceed a dose-dependent threshold, beyond which the benefit drops sharply. However, a second nivolumab application, within 7–10 days after the first, can extinguish a pathogen which the first nivolumab application failed to remove. The utility of increasing nivolumab total dose above 6 mg/kg is contingent on the patient's personal immune attributes, notably, the reinvigoration rate of exhausted CTLs and the overall suppression rates of functional CTLs. A baseline pathogen load, higher than 5,000 CFU/μL, precludes successful nivolumab and meropenem combination therapy, whereas when the initial load is lower than 3,000 CFU/μL, meropenem monotherapy suffices for removing the pathogen.

**Discussion:** Our study shows that early administration of nivolumab, 6 mg/kg, in combination with antibiotics, can alleviate bacterial sepsis in cases where antibiotics alone are insufficient and the initial pathogen load is not too high. The study pinpoints the role of precision medicine in sepsis, suggesting that personalized therapy by ICBs can improve pathogen elimination and dampen immunosuppression. Our results highlight the importance in using reliable markers for classifying patients according to their predicted response and provides a valuable tool in personalizing the drug regimens for patients with sepsis.

## Introduction

Bacterial sepsis is a severe life-threatening systemic dysregulated pro-and anti-inflammatory response to infection, often resulting in tissue damage, multiple organ dysfunction, and ultimately death (1). Recent global estimates of bacterial sepsis epidemiology report 48·9 million cases of sepsis and 11 million sepsis-related deaths in 2017, representing 19·7% of all deaths worldwide. Despite the global trend of decreasing sepsis burden, progress in the treatment of sepsis has been modest (2–4). An immediate administration of broad-spectrum antibiotics is the first-line treatment for the improvement of patient outcomes and reduction of mortality and morbidity due to sepsis (5–8). Efforts were also made to avoid hyper-inflammation, which characterizes the early stage of this disorder, by administering anti-inflammatory agents, including toll-like receptor (TLR) antagonists, anti-cytokine therapies, and corticosteroids (3). Disappointingly, these have often failed in relieving the septic condition (9). Patients who endure the initial phase of hyper-inflammation frequently enter a second, lengthier phase of immunosuppression, characterized by immune cell depletion and changes in receptor expression patterns, usually resulting in the acquisition of nosocomial infections, and often death (10, 11). There is an urgent need, therefore, to improve sepsis therapy by minimizing the duration of the immunosuppressive state, or preventing it altogether.

In cancer, cytotoxic T lymphocytes (CTLs) expand extensively upon encountering foreign antigens (12). Following antigen clearance and the resolution of the inflammation, the programmed cell death protein 1 (PD-1) receptors on the surface of CTLs bind to their ligands, PD-L1 and PD-L2, to generate a co-inhibitory signal, which suppresses the CTL expansion (13). Cancer cells hijack this natural self-constraining mechanism. By expressing the same co-inhibitory signal, they stimulate CTLs to undergo exhaustion, weakening the immune response prematurely and hampering cancer cell clearance (12, 14). The recently developed immune checkpoint blockers (ICBs) can counteract this cancer-induced ligand-receptor association, enabling reinvigoration of exhausted CTLs, restoration of anticancer immunity and suppression of cancer growth (15). As PD-1 and PD-L1 are upregulated in septic patients, it is plausible that ICBs, which were developed as oncology drugs, can also be suitable for the treatment of sepsis, preventing the critical immunosuppression phase and overcoming its often-lethal consequences (16, 17). Several ICBs, studied in murine models of sepsis, show significant effects on restoration of T cell function, reduction of inflammation, and improvement of survival (18). Recent clinical studies of the PD-1 inhibitor antibody, nivolumab (opdivo®), first approved for the treatment of melanoma, demonstrated favorable safety and tolerability in the treatment of septic patients (19, 20). However, excessive inflammation and a constellation of toxicities could still emerge under this immunotherapy (21–25), which is one reason successful clinical trials for ICBs in sepsis are still scarce. Possible adverse events include hepatitis, pneumonitis, enterocolitis and grade 3 anemia (26–28). Judicious use of ICB therapy, and careful regimen planning, based on assessment of personal benefits and risks for the selected agent(s), is therefore of utmost necessity.

Mathematical modeling can help disentangle the dynamic interactions between the pervading pathogen, the host cellular immunity and the drug. To construct a mathematical model, one makes simple assumptions about the major forces in the system, and formalizes them by the succinct language of mathematics. This enables *in silico* simulations of the system's behavior under different administration schedules of the drug(s), hence predicting the patient response to each application regimen. Models of this kind have proven useful for this purpose in a wide range of medical fields, including cancer immunotherapy by ICBs (29, 30). In sepsis, previous mathematical modeling has focused mainly on the shift of equilibrium between the pro- and anti-inflammatory signaling cascades, not considering the immunosuppressive arm (31, 32). Therefore, it was necessary to develop a model of sepsis-associated inflammation that would include potential drivers and inhibitors of immunosuppression.

To achieve this goal, Gillis et al. (33) devised “skeletal” mathematical models for bacterial sepsis, formalizing putative mechanisms which govern sepsis-associated inflammation and immunosuppression. Model simulations show that when no pathogen-induced CTL exhaustion is assumed, the immune system can permanently eliminate mild pathogens, while moderate and aggressive pathogens recover concurrently with the cellular immune arm, and by that stimulate another wave of immune reaction. In contrast, simulations of a model that includes the effect of exhaustion show progressively decreasing CTL counts and chronic bacteremia. In the latter scenario, administration of an ICB in combination with antibiotics can lead to pathogen clearance, if the ICB is applied sufficiently early (33).

For proceeding toward the implementation of sepsis immunotherapy in the clinical practice, we aimed to develop an optimization scheme for singling out treatment strategies for bacterial sepsis, which attain maximum efficacy with minimum adverse events. The scheme we developed relied on a general approach for optimizing drug schedules by use of mathematical mechanistic models, developed by Agur et al. (34). At the core of the method lies a new mathematical mechanistic model, formalizing the dynamic interactions in the drug-host-pathogen system. The new model–an extension of the much simplified models in (33)–was used to evaluate which treatments by the carbapenem antimicrobial agent meropenem, as the representative antibiotics (7) and the PD-1 blocker nivolumab (20), as the ICB under examination, could optimize bacterial sepsis therapy. We combined the extended disease model with the newly developed pharmacokinetics (PK) and pharmacodynamics (PD) models of the chosen drugs and simulated the combined model within the optimization scheme we had developed. Doing so, we could suggest improved regimens for the drugs used. Due to criticality of the sepsis condition, we chose short-term pathogen elimination (6 weeks) as the primary endpoint in our study (35).

## Methods

### The Disease Model

**Figure 1:**
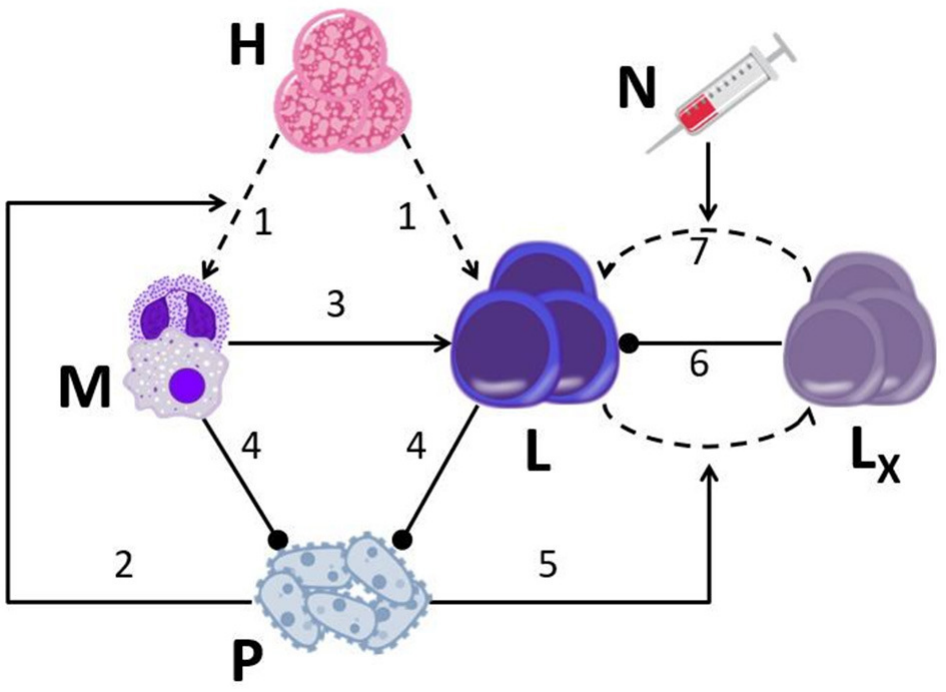
A graphical display of the drug-disease-host model. The model is based on seven major assumptions: **(1)** HSCs (H) continually differentiate into myeloid cells (M; neutrophils, macrophages) and lymphoid cells (L, CTLs); **(2)** the presence of pathogen (P) biases HSC differentiation toward the myeloid lineage; **(3)** CTLs encounter APCs (such as macrophages) that have phagocytized antigen, and expand their population in response; **(4)** neutrophils and CTLs inhibit pathogen growth; (**5)** pathogen causes healthy CTLs (L) to differentiate into exhausted CTLs (L_X_) (via activation of the programmed cell death-1 receptor; PD-1); **(6)** exhausted CTLs hinder proliferation of healthy CTLs; **(7)** nivolumab (N) induces the reinvigoration of exhausted CTLs back into the functional CTL compartment by blocking the PD-1 pathway. Straight arrows, activation; dashed arrows, differentiation; blunted arrows, inhibition.

Our disease model, described in Figure 1, is an extension of the one described in Gillis et al. (33). In the current embodiment, we took explicit account of the exhaustion process and the reinvigoration of exhausted CTLs. Since sepsis induces a systemic, multi-organ failure, the model's descriptions of the interactions between the pathogen and the immune system of the host, are not limited to a specific organ or tissue, which is to say the model is non-spatial. Rather, the two hematopoietic arms, myeloid and lymphoid, representing the innate and adaptive immune systems, respectively, are taken as the core of these interactions. In our model, hematopoietic stem cells (HSCs) differentiate into each one of the two hematopoietic arms with complementary probabilities, *a*_*M*_ for myeloid and 1 − *a*_*M*_ ≡ *a*_*L*_ for lymphoid. We assumed that the probability of HSCs differentiating into myeloid cells increases with the presence of pathogen, because of the increasing demand for blood neutrophils. This effect is termed emergency granulopoiesis (36). According to our model, myeloid cells encourage the proliferation of CTLs, while the pathogen depletes them by upregulating the PD-1/PD-L1 pathway, leading to CTL exhaustion, directly reducing their impact (37). Although the role of antibody-producing B cells in sepsis, serving as gatekeepers of bacterial infection (38), is more important than previously thought, for the sake of parsimony, B cells and antibodies are neglected in our model, CTLs being the focus of our study in optimizing sepsis therapy.

We also allowed for the possibility that the pathogen can indirectly suppress CTLs' proliferation, by exhausting functional CTLs. Exhausted CTLs can hamper the proliferation of functional T cells, e.g., by releasing anti-inflammatory cytokines, such as IL-10 and TGF-β (39), or by suppressing the expression of stimulatory receptors and ligands, such as Human Leukocyte Antigen DR isotype (HLA-DR), by antigen presenting cells (APCs) (40, 41). In our model, administration of ICBs reinvigorates CTLs that are in the process of exhaustion, returning them to the compartment of functional CTLs (42). Moreover, the model pathogen grows according to a logistic function, all the while being suppressed by neutrophils and CTLs. The equations representing these processes are given below here (all cells are counted in a volume of 1 μL).

(1) $$\begin{array}{r} \dot{M}=f_{H}\;(P)\;\cdot a_{M}H-\mu_{M}M. \end{array}$$

(2) $$\begin{array}{l} \dot{L}=\left(1-f_{H}\;(P)\;\cdot a_{M}\right)H+f_{AP}\left(\tilde{M}\right)\;\cdot r_{L}L\left(1-\frac{L}{K_{L}}\right)-\mu_{L}L\\ \;-f_{X}\;(P)\;\cdot L+f_{r}\;(N)\;L_{X}. \end{array}$$

(3) $$\begin{array}{r} \dot{L_{X}}=f_{X}\;(P)\;\cdot L-f_{r}\;(N)\;L_{X}. \end{array}$$

(4) $$\begin{array}{r} \dot{P}=r_{P}\;(A)\;\cdot P\left(1-\frac{P}{K_{P}}\right)-\kappa_{M}M\frac{P}{J_{P}+P}-\kappa_{L}L\frac{P}{J_{P}+P}. \end{array}$$

(5) $$\begin{array}{r} f_{H}\;(P)\;=\frac{P_{\alpha }+\alpha P}{P_{\alpha }+P}. \end{array}$$

(6) $$\begin{array}{r} f_{AP}\left(\tilde{M}\right)=\frac{s_{X}}{s_{X}+L_{X}}\cdot (1+\frac{\beta \tilde{M}}{J_{M}+\tilde{M}}). \end{array}$$

(7) $$\begin{array}{r} f_{X}\;(P)\;=\frac{\mu_{X}}{1+e^{-\gamma (P-P_{\gamma })}}. \end{array}$$

(8) $$\begin{array}{r} f_{r}\;(N)\;=q_{X}\cdot \frac{N}{N+N_{50}}. \end{array}$$

In Eqs 1–8, *M* is the number of cells in the myeloid compartment. Neutrophils are by far the most abundant of these (43), and we therefore evaluated the associated parameters accordingly. For this reason, we refer to the variable *M* as “neutrophils” when presenting our simulations in the Results section. The variable *L* is the number of CTLs; *L*_*X*_ denotes the number of exhausted CTLs; *P* is the number of pathogens; *f*_*H*_ (*P*) is the HSC differentiation skew function; *f*_*AP*_(*M*) is the function expressing the rate of antigen presentation by myeloid cells; *f*_*X*_(*P*) is the exhaustion rate function; *f*_*r*_(*N*) is the reinvigoration function; *N*, nivolumab (ICB) concentration. The parameter *N*_50_ from Eq. 8 is estimated according to Hotchkiss' findings on receptor occupancy, which show a sustained effect of nivolumab for the entire observed period of ~3 months, even when the drug concentration was significantly reduced. We therefore set this parameter at the relatively low level of 0.3 mg/kg, so that the drug's dose-efficacy function reaches saturation relatively quickly. We deliberately formulated the exhaustion function and the reinvigoration function not to be symmetric. *f*_*X*_(*P*) is a sigmoid function, such that if *P* ≪ *P*_γ_ the exhaustion effect is quite weak (39). This is to reflect the observation that the onset of exhaustion due to PD-1 binding is a gradual event, induced by a persistently high pathogen load (over 10^3^ CFU/μL). In contrast, the pharmacodynamic effect of nivolumab, measured in clinical trials as receptor occupancy, behaves as a hyperbolic function of the dose, hence the formulation of *f*_*r*_(*N*) (19).

The parameters in Eqs 1–8 are: *H*, HSC population size; *a*_*M*_, probability of HSC differentiation into a myeloid cell; μ_*M*_, neutrophil death rate; *r*_*L*_, CTL proliferation rate; *K*_*L*_, maximum CTL number; *μ*_*L*_, CTL death rate; *r*_*P*_(*A*), pathogen growth rate, dependent on *A*, antibiotics concentration; *K*_*P*_, maximum pathogen load; κ_*M*_, pathogen killing rate by neutrophils; κ_*L*_, pathogen killing rate by CTLs; *J*_*P*_, pathogen load which induces half-maximal pathogen elimination; *P*_α_, regulating pathogen load for *f*_*H*_; *α*, maximum skew of HSC differentiation into myeloid lineage; *β*, immunogenicity parameter, representing magnitude of antigen presentation by APCs to CTLs; $\tilde{M}$, neutrophil number above homeostasis (see below); *J*_*M*_, myeloid number which induces half-maximal antigen presentation by APCs to CTLs; *s*_*X*_, exhausted cell number which induces half of maximal CTL suppression (indirect suppression)^1^; *μ*_*X*_, maximum CTL exhaustion rate; *γ*, steepness of exhaustion as function of pathogen; *P*_γ_, half maximum pathogen load for *f*_*X*_; *q*_*X*_, reinvigoration rate due to nivolumab; *N*_50_, half maximum nivolumab concentration for *f*_*r*_. For further details on the parameters and their estimation, see Table 1.

**Table 1:** Parameter names and values in model equations, including modes of evaluation and sources.

**Parameter**	**Description**	**Equation**	**Value**	**Units**	**References**
*H*	HSC supply	1	0.2	10^3^ cells/(μL*h)	(44)
*M*_0_	Neutrophil level at homeostasis		4.5	10^3^ Cells/μL	(45)
*L*_0_	CTL level at homeostasis		1.5	10^3^ Cells/μL	(45)
*a*_*M*_	Probability of HSC differentiation into myeloid cells	1	0.4	1	(46) Adjusted
μ_*M*_	Neutrophil death rate	1	0.02	1/h	Calculated from steady state (homeostasis)
*r*_*L*_	CTL proliferation rate	2	1	1/h	Adjusted
*s*_*X*_	Exhausted CTL level which induces half-maximum CTL suppression	6	5	10^3^ cells/μL	Personal
μ_*X*_	CTL exhaustion rate, asymptotic in *P*	7	0.01	1/h	Personal
*K*_*L*_	Maximum CTL levels	2	20	10^3^ Cells/μL	Adjusted
μ_*L*_	CTL death rate	2	0.4	1/h	Calculated from steady state (homeostasis)
*r*_*P*_	Pathogen growth rate	4	Dynamic	1/h	(47)
*K*_*P*_	Maximum pathogen load	4	20	10^3^ Cells/μL	(48)
κ_*M*_	Pathogen killing rate by neutrophils	4	0.02	10^3^ CFU/(h*cells)	Adjusted
κ_*L*_	Pathogen killing rate by CTLs	4	0.08	10^3^ CFU/(h*cells)	Adjusted
*J*_*P*_	Pathogen load which induces half-maximal pathogen elimination	4	1	10^3^ CFU/μL	Adjusted
α	Maximum skew of HSC differentiation into myeloid lineage	5	2	1	Adjusted
*P*_α_	Controlling pathogen load for skew of HSC differentiation	5	1	10^3^ CFU/μL	Adjusted
β	Immunogenicity parameter, representing magnitude of antigen presentation by APCs to CTLs	6	1.2	1	Adjusted
*J*_*M*_	Myeloid level which induces half-maximal antigen presentation by APCs to CTLs		1	10^3^ Cells/μL	Adjusted
γ	Steepness of exhaustion as function of pathogen	7	10	10^−3^ μL/CFU	Adjusted
*P*_γ_	Pathogen load which induces half maximum CTL exhaustion	7	1	10^3^ CFU/μL	Adjusted
*q*_*X*_	Reinvigoration rate due to Nivolumab	8	0.2	1/h	Personal
*N*	Nivolumab concentration	8	Dynamic	mg/kg	(19)
*N*_5_0	Nivolumab concentration producing half of maximum effect	8	0.3	mg/kg	(19)
*C*_0_	Constant concentration of meropenem	9	70	mg/L	(49)
κ	Steepness of antibiotic PD function	9	1	1	(50)
*r*_*max*_	Maximum pathogen growth	9	0.8	1/h	(47)
δ_*max*_	Maximum pathogen elimination by meropenem	9	−0.2	1/h	(47)
*MIC*	Meropenem minimum inhibitory concentration	9	100 (pathogen-specific)	mg/L	(50)
*N*_1_	Initial nivolumab concentration	10	4	mg/kg	(19)
*N*_2_	Equilibrium nivolumab concentration	10	2	mg/kg	(19)
λ_1_	Initial nivolumab clearance	10	0.02	1/h	(19)
λ_2_	Equilibrium nivolumab clearance	10	0.002	1/h	(19)

### Drug Pharmacokinetics/Pharmacodynamics

#### Meropenem

In this work, we modeled the carbapenem antimicrobial agent, meropenem, as the representative antibiotics (7). For simulating realistic administration regimens of this drug, we chose to model a continuous i.v. administration of meropenem. This is because many physicians today prefer continuous application, to limit the risk of emergence of resistant pathogens under intermittent infusion of this short half-life drug (51). This is the case even though the superiority of either continuous i.v. administration or intravenous bolus administration of meropenem for patients with sepsis is still debated (6, 52). For modeling continuous i.v. administration of meropenem, the drug concentration was described as *A*(*t*) = *C*_0_, with *C*_0_ being the constant meropenem concentration introduced by the injection. Here *A*(*t*) is the serum meropenem concentration at time *t*. The value of *C*_0_ was taken from (6, 8) and was set to 70 mg/L. The PD effects on the pathogen were formulated as a Hill function, as proposed by Regoes et al. (47):

(9) $$\begin{array}{r} r_{P}\left(A\right)=r_{\max}-\frac{\left(r_{\max}+\delta_{\max}\right){\left(\frac{A}{MIC}\right)}^{\kappa }}{{\left(\frac{A}{MIC}\right)}^{\kappa }+\frac{\delta_{\max}}{r_{\max\;}}}. \end{array}$$

In Equation 9, *r*_*P*_(*A*) is the pathogen's growth rate as a function of *A* (see Eq. 4 above); *r*_max_ is the maximum pathogen growth rate; δ_max_ is the maximum pathogen elimination rate by antibiotics; *MIC* is the minimum inhibitory concentration (MIC); κ is the steepness of the Hill function (see Table 1 for further details). Since the MIC depends on the specific bacteria (8), we chose to model those bacteria, which have a large enough value of MIC to persist through antibiotic monotherapy, as otherwise there is no *a priori* purpose to examine the antibiotics and ICB combination. In recent decades, a growing number of bacterial strains have developed significant resistance capabilities to various antimicrobial therapies (53). This is another motivation for our choice of MIC value.

#### Nivolumab

We modeled nivolumab (opdivo®), a programmed cell death-1 (PD-1) pathway inhibitor hitherto used in oncology, as the representative ICB, exerting its effect on the reinvigoration rate of exhausted CTLs (Figure 1). Hotchkiss et al. (19) performed a Phase Ib trial, testing the toxicity of nivolumab in patients with sepsis. The results of this trial did not reveal any unexpected safety findings, nor did it report any drug-related severe adverse events, or evidence for “cytokine storm” in patients. Moreover, in another study by Watanabe et al., the toxicity in exposure to a single dose of nivolumab, 960 mg, was comparable with that of nivolumab, 3 mg/kg every 2 weeks, in the oncological setting (54). Since Hotchkiss et al.'s trial had a larger sample size, we chose to model the PK/PD dynamics according to their study.

The nivolumab serum concentrations therein displayed a predictable PK profile and dose-related increases in exposure. This profile is typical of a two-compartment PK model—blood and well-perfused organs being the central compartment and poorly perfused organs and tissues being the peripheral compartment. Accordingly, a good approximation of the serum drug concentration is

(10) $$\begin{array}{r} N(t)=N_{1}e^{-\lambda_{1}t}+N_{2}e^{-\lambda_{2}t}, \end{array}$$

where, *N*_1_ + *N*_2_ amounts to the maximal concentration of the drug; *N*_1_ is the initial concentration; *N*_2_ is the equilibrium concentration; λ_1_ is the initial nivolumab clearance; λ_2_ is the equilibrium nivolumab clearance. We estimated *N*_1_, *N*_2_, λ_1_, λ_2_ according to the PK parameters in Hotchkiss' study (19), and by fitting the concentrations, simulated using equation (10) to the observed ones (goodness of fit being, *R*^2^ = 0.87). The parameter estimations appear in Table 1 and explained below.

In our model, the PD effect of the ICB drug is expressed as a hyperbolic function: $f_{r}\left(N\right)=q_{X}\frac{N}{N+N_{50}}$ (see Eqs 2, 3, 8). The parameter *N*_50_ from Eq. 8 is estimated according to Hotchkiss' findings on receptor occupancy, which show a sustained effect of nivolumab for the entire observed period of ~3 months, even when the drug concentration was significantly reduced. We therefore set this parameter at the relatively low level of 0.3 mg/kg, so that the drug's dose response function reaches saturation relatively quickly.

### Parameter Estimation

Table 1 contains the meanings and values for the model's parameters. For those parameters with a definite value found in the scientific literature, we assigned that value. Parameters which are noted as “adjusted” were estimated by calculation of their necessary values for the model variables' steady states and simulated ranges to be clinically plausible (those variables being neutrophils, CTLs, pathogen).

### Treatment Optimization

In order to evaluate the overall benefit of different treatment schedules, we formulated an *optimization problem*, which would reflect the various goals of the treatment, according to criteria set by the physician. In general, these criteria may be, for example, time to reach a specified disease state, adverse effects, quality of life, cost of treatment, etc. Setting the optimization problem, we formalized the associated *fitness function*, namely, the objective function that would be used to summarize, as a single figure of merit, how good a given design solution is in achieving the treatment goals according to the set criteria. Subsequently, all the potential design solutions—in our case, the potential treatment schedules and potential personal parameters—were tested by local search heuristics to find a solution, e.g., a specific treatment regimen for a specific patient, which would locally optimize the fitness function (34). We visualized the results in *fitness landscapes*, presented as color-schemed heat maps.

In this work, we addressed the problem of finding the combined schedule of meropenem and nivolumab, which would best (i) delimit the overall pathogen load and (ii) eliminate the pathogen as early as possible, while (iii) maintaining a sufficiently high level of functioning CTLs, to minimize immunosuppression and maintain the adaptive immune system ready for further pathogen invasions. We set the associated criteria to be

- Total pathogen load over time, *∫* **P(t)dt**.
- Time until the pathogen is eliminated, **T**_**cure**_.
- CTL suppression level, **Z_L_= *∫* I_L<L_0__·(L_0_−L(t))dt**, where *L*_0_ is CTL level at homeostasis, and *I*_*L*<*L*_0__ is an identity function which returns 1 if indeed *L* < *L*_0_ and 0 otherwise (Table 1).

The rationale for including these measurements in the optimization function is that both high pathogen load and prolonged infections can cause damage to the patient. We wished to consider also cases where the pathogen load is relatively low, yet persistent, which can, for example, lead to catastrophic outcomes, such as multiple organ failure (MOF), due to chronic inflammation (3, 55). We included CTL suppression level, *Z*_*L*_, to represent the potency of the cellular immune system, and to reflect its ability to suppress secondary infections, which can frequently occur in an intensive care unit (ICU) setting (56, 57). Additionally, *Z*_*L*_ measures the efficacy of the drug in achieving its specific purpose of reinvigorating CTLs. All the measurements are weighted by coefficients in accordance with the importance the doctor would wish to give to each of them in specific cases. Integrating the different criteria into one formula, we arrive at the following scalar-valued fitness function

(11) $$\begin{array}{r} F_{w}\left(S\right)=\frac{1}{C}(\theta -\alpha_{w}\int P\left(S\right)-\beta_{w}T_{cure}\left(S\right)-\gamma_{w}Z_{L}\left(S\right)), \end{array}$$

where *F*_*w*_ is the fitness associated with the specific weighting *w* given by the function's coefficients, and *θ* is a normalization factor, mapping the function's range to the interval between 0 and 1, with 1 being the optimum. The fitness *F* receives as input the assessed nivolumab administration schedule *S*, a 2 by *k* matrix, *k* being the number of nivolumab applications. The first row of *S* is administration times and its second row is dose (the administration of meropenem remains the same in all shown experiments). Note that in this study, we only simulated *k* = 1, 2 due to the characteristic short time-span of the pathology we examined. Both *∫ P* and *Z*_*L*_ are in units of population size per 1 μL of blood, while *T*_*cure*_ is given in units of days. In the current work we chose the weights *α*_*w*_ = 1, *β*_*w*_ = 21, *γ*_*w*_ = 3. In this way, *∫ P* and *T*_*cure*_ have a comparable influence on the value of *F*_*w*_, while *Z*_*L*_ has a slightly lower influence, given our units of choice. Further changes in these coefficients can modulate the importance of each element in the fitness function according to the requirements of the treating physicians.

### Simulations and Analysis

We performed all simulations and analysis using MATLAB 2016a and the statistical package RStudio^©^.

## Results

**Figure 2:**
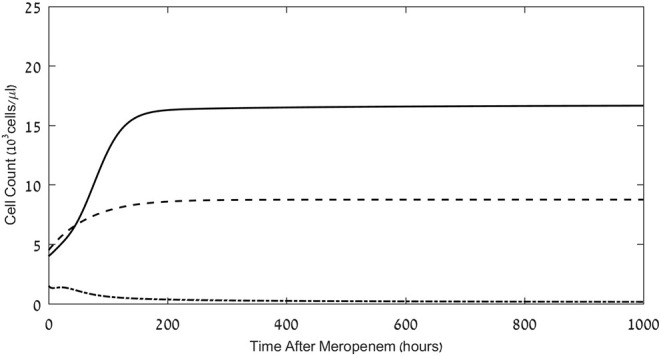
Effects of meropenem application on the pathogen load and neutrophil and CD8^+^ CTL counts. Model simulations of the three populations of interest: neutrophils (dashed lines), CD8^+^ CTLs (dash-dot) and pathogen (continuous), under continuous treatment with meropenem (antibiotics), 70 mg/L i.v., administered at *t* = 0. Initial neutrophil level *M*(*t* = 0) = 4.5·10^3^Cells/μL; initial CTL level *L*(*t* = 0) = 1.5·10^3^Cells/μL. Initial pathogen load *P*(*t* = 0) = 4·10^3^ CFU/μL. For equations see **Methods** section. For parameter values see Table 1.

**Figure 3:**
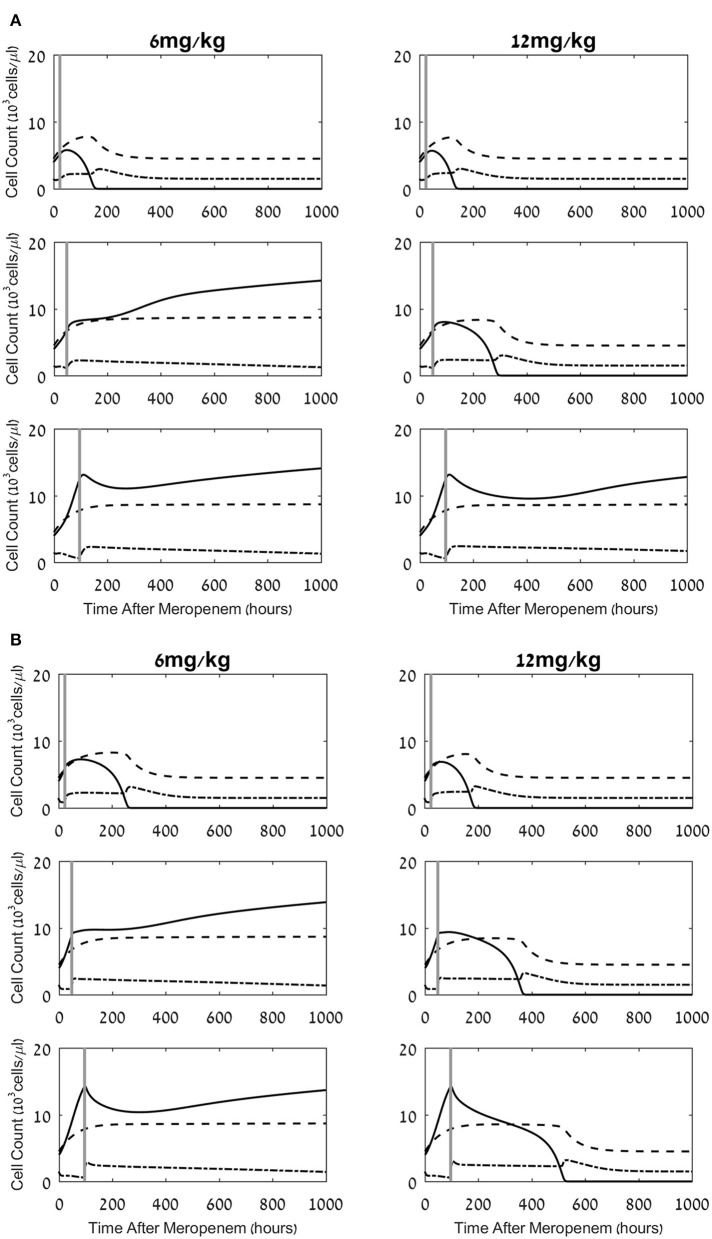
Effects of a combined meropenem and nivolumab regimen on the pathogen load and the neutrophil and lymphocyte cell levels. **(A,B)** Model simulations of the three populations of interest: neutrophils (dashed lines), lymphocytes (dash-dot) and pathogen (continuous), under continuous treatment with meropenem (antibiotics), 70 mg/L i.v. application, administered at *t* = 0, and nivolumab, single dose, 6 mg/kg, 12 mg/kg (left column, right column, respectively) administered at 24 h, 48 h, 96 h (top, middle, bottom row, respectively; vertical gray lines). Parameters for **(A)** are CTL exhaustion rate *μ*_*X*_ = 0.01h^−1^; CTL suppression by exhausted CTLs $s_{X}^{*}=45\cdot 10^{3}$Cells/μL. Parameters for **(B)** are CTL exhaustion rate *μ*_*X*_ = 0.07h^−1^; CTL suppression by exhausted CTLs $s_{X}^{*}=5\cdot 10^{3}$Cells/μL. Initial neutrophil level *M*(*t* = 0) = 4.5·10^3^Cells/μL; initial CTL level *L*(*t* = 0) = 1.5·10^3^Cells/μL. Initial pathogen load *P*(*t* = 0) = 4·10^3^ CFU/μL. For equations see methods section. For parameter values see Table 1.

**Figure 4:**
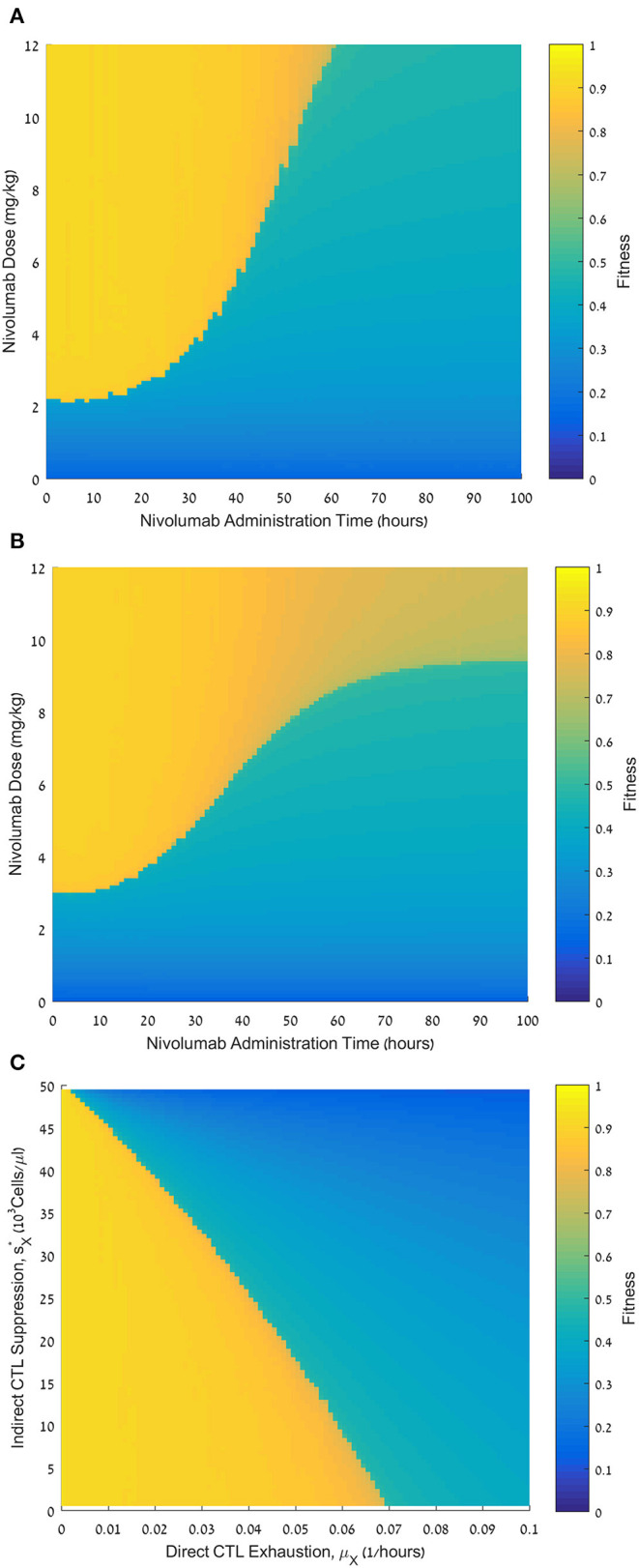
Fitness of varying nivolumab regimens and the effect of personal parameters. **(A)** Fitness values, *F*, for nivolumab administration times ranging from 0 to 100 h (abscissa) and nivolumab doses ranging from 0 to 12 mg/kg (ordinate). CTL exhaustion rate μ_*X*_ = 0.01 h^−1^; CTL suppression by exhausted CTLs $s_{X}^{*}=45\cdot 10^{3}$CFU/μL. **(B)** Fitness values, *F*, for nivolumab administration times ranging from 0 to 100 h (abscissa) and nivolumab doses ranging from 0 to 12 mg/kg (ordinate).; CTL suppression by exhausted CTLs $s_{X}^{*}=5\cdot 10^{3}$CFU/μL. **(C)** Fitness values, *F*, for CTL exhaustion rate, μ_*X*_, ranging from 0 to 0.1 h^−1^ (abscissa), and CTL suppression by exhausted CTLs, $s_{X}^{*}$, ranging from 0 to 50·10^3^Cells/μL (ordinate). Nivolumab administration time is 48 h; nivolumab dose is 6 mg/kg (ordinate). Initial pathogen load $P_{0}=4\cdot 10^{3}$CFU/μL (all plates). For equations see methods section. For other parameter values see Table 1. Note that $s_{X}^{*}=50\times 10^{3}Cells/\mu l-s_{X}$, where *s*_*X*_ is the suppression parameter in Eq. 6 (the motivation for using this transformation is to make the graph more understandable, since *s*_*X*_ itself has an inverse relationship with the suppression rate).

**Figure 5:**
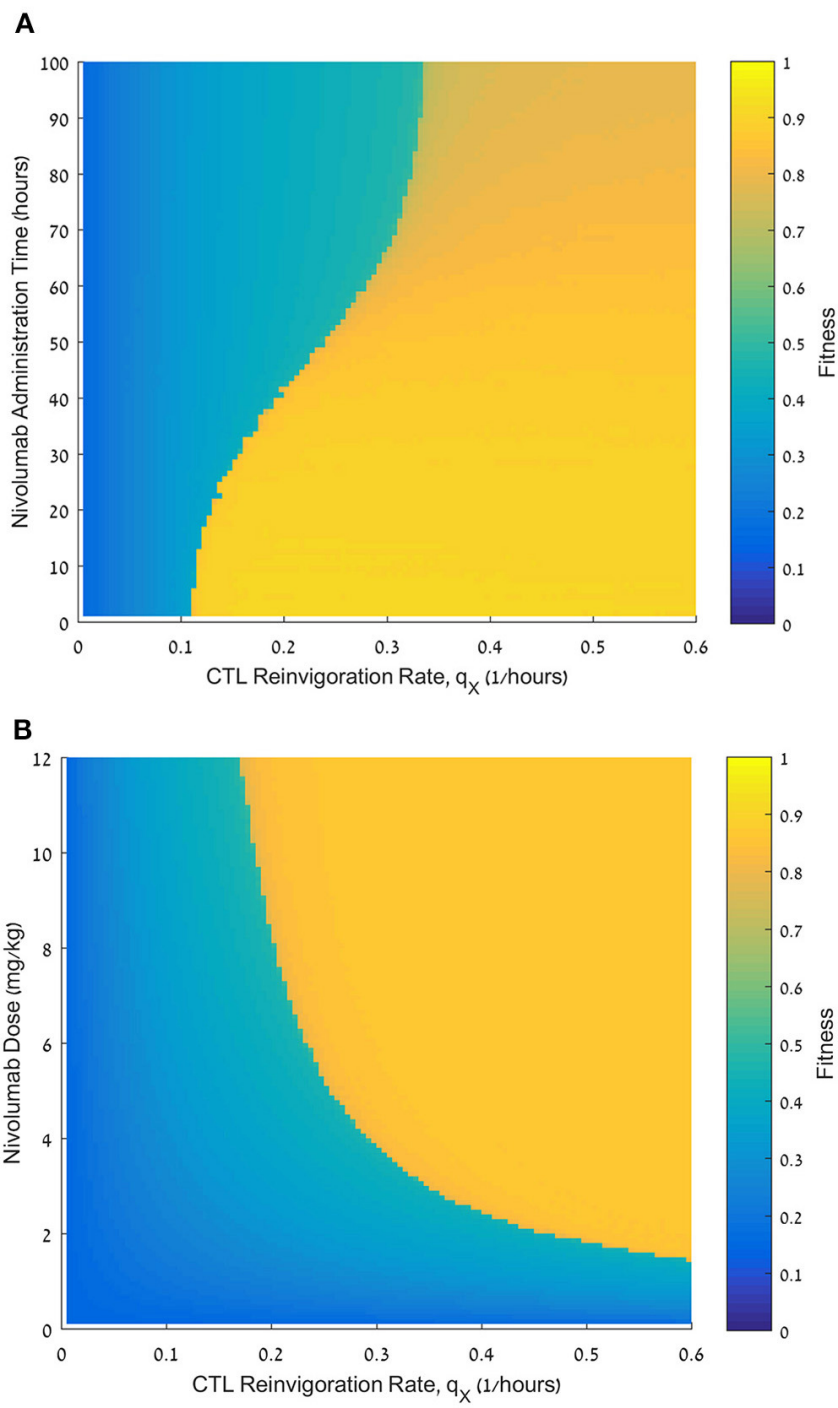
Effect of the reinvigoration rate on the fitness of varying nivolumab regimens. **(A)** Fitness values, *F*, for reinvigoration rates *q*_*X*_, ranging from 0 to 0.6 h^−1^ (abscissa) and nivolumab administration times ranging from 0 to 100 h (ordinate), nivolumab dose *D* = 6 mg/kg. **(B)** Fitness values, *F*, for reinvigoration rates *q*_*X*_, ranging from 0 to 0.6 h^−1^ (abscissa) and nivolumab doses ranging from 0 to 12 mg/kg (ordinate); nivolumab administration time *t*_*N*_ = 48 h. Initial pathogen load $P_{0}=4\cdot 10^{3}$ CFU/μL (both plates). For equations see **Methods** section. For other parameter values see Table 1.

**Figure 6:**
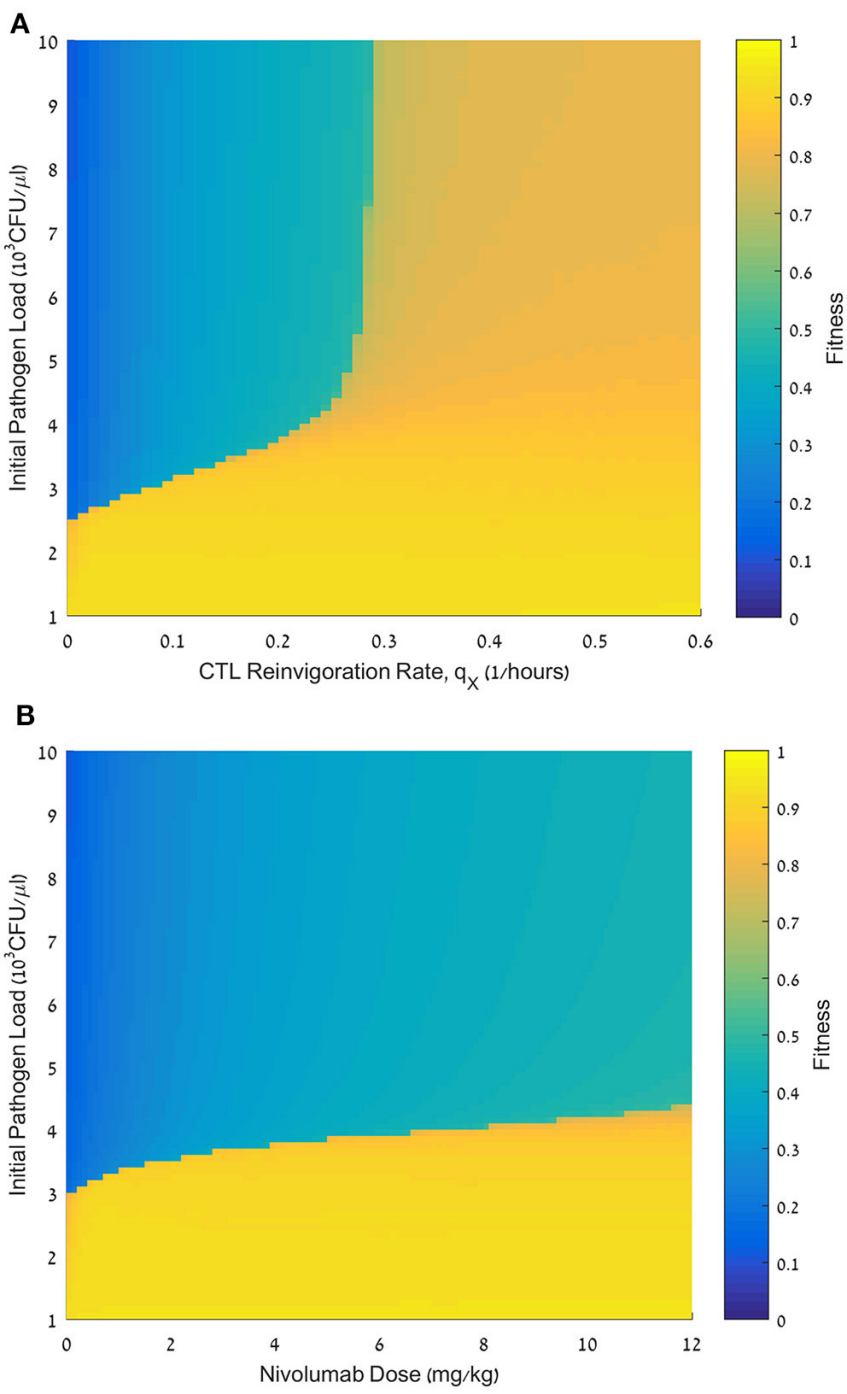
Effect of initial pathogen load on the fitness of nivolumab regimens. **(A)** Fitness values, *F*, for initial pathogen loads ranging from 0 to 10·10^3^ CFU/μL (ordinate) and reinvigoration rates, *q*_*X*_, ranging from 0 to 0.6 h^−1^ (abscissa). nivolumab administration time *t*_*N*_ = 48 h; dose *D* = 6 mg/kg. **(B)** Fitness values, *F*, for initial pathogen loads ranging from 0 to 10·10^3^ CFU/μL (ordinate), and nivolumab dose, *D*, ranging from 0 to 12 mg/kg. For equations see methods section. For other parameter values see Table 1.

We numerically simulated the combined disease/drugs model over a period of 1,000 h (~ 6 weeks), taking account of the three populations of interest, which constitute the model variables: pathogen, CTLs and neutrophils (*P, L, M* in the equations; see methods section). All the results brought forward below, and depicted in Figures 2–6, reflect the simulations of the same, relatively aggressive, bacterial pathogen, the antibiotic meropenem, applied via continuous i.v. infusion inducing plasma concentrations of 70 mg/L (58) with, or without, a single administration of the ICB, nivolumab, either 6 mg/kg or 12 mg/kg. These family of regimens stand in accordance with the doses used so far in clinical trials for ICBs in sepsis (19, 20, 54). We also tested the benefit in multiple nivolumab dosing, either by fractionation, or by multiplication of a reference dose. In these scenarios, we administered nivolumab once again, seven or 10 days after the first application of this drug, total dose equaling or doubling that of a single administration. All simulations begin at the onset of antibiotics application (*t* = 0).

### Antibiotics Combined With a Single Application of ICB Can Be Sufficient for Pathogen Elimination When Antibiotics Alone Fail

As observed in the results depicted in Figure 2, the simulated treatment by meropenem alone does not lead to elimination of the pathogen, or to prevention of the pathogen-associated severe CTL depletion, when *P*_0_, the pathogen load at *t* = 0, is 4 × 10^3^ CFU/μL (for other parameters, see caption to Figure 2). Already early in treatment, the persistent pathogen succeeds in forcing the CTL levels to decrease, and by that triggers an unmanageable rise in pathogen load until it reaches its system-determined carrying capacity. Note that in most cases, an infection of this severity would lead to death well before the end of the simulated timeline. However, in this specific system, the application of the ICB, nivolumab, 6 mg/kg or 12 mg/kg, 24 h following the antibiotic treatment, extinguishes the pathogen within <1 week, and sets off a slow return of the immune system toward homeostasis (Figure 3A top row). Comparing the top row images in Figure 3A to Figure 2, one notices that the contribution of the immunotherapeutic agent is to keep CTL count sufficiently high for long enough as to force a continuous decrease in pathogen load until its complete elimination. Once this occurs, immune cells return to homeostasis levels. One can see in the top row images in Figure 3A that the effects of a single nivolumab application, at 24 h following the onset of meropenem infusion, are almost the same when the dose of 6 mg/kg is doubled. As is shown hereafter, this will not be so when nivolumab application is delayed.

Changing the time of nivolumab administration, from 24 h to 48 h or 96 h following antibiotics, can yield radically different results. While in all the cases shown in Figure 3A nivolumab administration temporarily relieved CTL depreciation, the treatment did not necessarily lead to a beneficial result for the patient, that is, pathogen elimination within 6 weeks. When nivolumab was administered at 48 h, pathogen elimination was achieved by the 12 mg/kg dose, but not by the smaller dose, 6 mg/kg. In the latter case, the increase in CTL levels due to the drug was not sufficient for bending down the non-decreasing pathogen growth curve. One can also note that when 6 mg/kg were applied at 48 h, the CTL counts gradually decreased (Figure 3A, middle row) and continued to decrease below the life-risking threshold of 10^3^ cells/μL (59) 4 weeks after treatment onset (not shown). In our simulations, when nivolumab was applied at 96 h, the pathogen was already too widespread to be removed by the CTLs, even if nivolumab was administered at the higher dose of 12 mg/kg and the CTL levels increased due to reinvigoration (Figure 3A, bottom row). At this time, the pathogen grew unchecked while also inducing CTL exhaustion. Comparison of this result to that of nivolumab administration at 48 h—which only succeeded with the larger dose—indicates that in the simulated system, the maximum interval between meropenem and nivolumab dosing, for successful pathogen elimination, depends on the ICB dose. However, as the 96 h application implies, the inter-dosing interval cannot be stretched too far. Overall, these results point to a dose-dependent maximum meropenem-nivolumab inter-dosing interval for successfully applying an ICB drug. In contrast, Figure 3B, displaying simulation results for the same regimens as Figure 3A, with different personal immune-related parameters (i.e., CTL exhaustion rate and CTL suppression by exhausted cells), shows that for some patients, increased nivolumab doses can lead to favorable results, in terms of pathogen elimination, even with application at 96 h (see also Figure 4B below).

#### The Threshold Effect

To fully assess the potential effects of the nivolumab dose and the permissible time interval for its administration after the onset of antibiotic infusion, we simulated the model over a relatively large spectrum of nivolumab doses and administration times, evaluating the fitness (i.e., benefit) of each treatment regimen by the fitness function (Eq. 11). As seen in Figure 4A, the fitness landscape displays a clear threshold effect: sufficiently large doses of nivolumab had maximum benefit if the inter-dosing interval between meropenem and nivolumab did not exceed a certain limit. Above this limit, the treatment benefit dropped sharply. However, we observe that higher doses of nivolumab maintained a high fitness over somewhat longer time windows, and, in these cases, the transition from maximum to minimum fitness schedules was somewhat less abrupt.

### Personal Immune Parameters Determine the Benefit of the Applied Nivolumab Schedule

#### Effect of CTL Exhaustion Rate and Suppression of Effector CTLs by Exhausted Cells

In our model, we postulated direct and indirect mechanisms, reducing the number of effector CTLs. First, the pathogen directly reduces the effector CTL levels (**L**) by sending effector CTLs into the exhausted compartment (**L**_**X**_; Figure 1, arrow 5). We assumed that this occurs according to a sigmoid function at a maximum rate, *μ***_X_** (**Methods**, Eq. 7). Second, we examined the possibility that exhausted CTLs themselves can hinder the expansion of the functional CTLs (Figure 1, arrow 6). In our model, this is controlled by the parameter $s_{X}^{*}=50\times 10^{3}Cells/\mu l-s_{X}$ (see **Methods**, Eq. 6 and Figure 1, arrow 6). We examined the effects of these assumptions on the benefit of the combined treatment. In Figure 4B, we present another administration landscape like Figure 4A, but for a virtual patient with different values for these parameters. For this patient, the direct suppression (*μ***_X_**) is significantly higher than the one examined in Figure 4A, while the indirect suppression ($s_{X}^{*}$) is substantially lower. Results in Figure 4B show a similar threshold effect to that in Figure 4A. However, in the scenario studied in Figure 4B, the application of large doses (over 9 mg/kg) shows no pronounced “drop” in fitness in terms of administration time, though a gradual decline is observed as the dosing interval is prolonged. This difference appears to capture a clear value in identifying the patient's susceptibility to the different suppression mechanisms in the model. The benefit of meropenem infusion with nivolumab, 6 mg/kg, applied at 48 h, for a range of personal suppression parameters, *μ*_*X*_ and *s*_*X*_, is shown in Figure 4C, exhibiting a roughly linear additive relationships between the two putative suppression mechanisms. This means that in our model, it is the overall suppression of CTL's replication which is significant, and not its different components. Furthermore, our results indicate that when exhaustion rate is relatively large, the treatment is non-beneficial even under no suppression of functional CTLs by exhausted cells.

#### Effects of the Reinvigoration Rate of Exhausted Effector CTLs

To test whether in sepsis, the personal reinvigoration rate, *q*_*X*_, can affect the response to nivolumab, we simulated patients with varying reinvigoration rates for a range of administration times of the immunotherapeutic drug, all with a standard nivolumab dose, 6 mg/kg. We then calculated the fitness, *F*, for each pair of reinvigoration rate and administration time, and drew the fitness landscape. Here too, the initial pathogen load was set at 4,000 CFU/μL (Figure 5A). Note that the fitness landscape was calculated with $\mu_{X},\;s_{X}^{*}$ values, which give roughly equal weight to direct exhaustion and indirect suppression of effector CTLs, corresponding to Figures 3A, 4A (see above). We see in Figure 5A that the reinvigoration rate, *q*_*X*_, plays a critical role in determining the benefit of the combined treatment protocol. Patients with low *q*_*X*_ receive little benefit from nivolumab administration regardless of timing. For patients with moderate *q*_*X*_, the effect is dichotomic, depending on the administration time: the fitness with early administration is much higher than that with late administration. In contrast, for patients with high *q*_*X*_, the decline in fitness due to increasing delay after meropenem is small and gradual, rather than dichotomic, indicating that patients with large reinvigoration rate are less susceptible to a delay in the ICB application.

We further examined the role of reinvigoration capacity, this time in terms of dose effect (Figure 5B). Here, we fixed nivolumab administration time to be 48 h following meropenem, and varied its administered dose, *N*, and the patient's reinvigoration rate, *q*_*X*_. Our results suggest that the larger the reinvigoration rate, the less nivolumab is required for pathogen elimination. In other words, there is a reciprocal relationship between both variables.

### Initial Pathogen Load Is a Telling Prognostic Marker

We next tested the dependence of the treatment benefit on the pathogen load, *P*_0_, at treatment initiation. Figure 6 shows the results of this analysis, varying *P*_0_ in conjunction with the patient's reinvigoration rate, *q*_*X*_ (Figure 6A), or dose (Figure 6B). Figure 6A shows a fitness landscape for a specific set of personal patient parameters. This landscape is divided into three subspaces. One subspace accounts for pathogen loads smaller than 3 × 10^3^ CFU/μL. Such loads can be eliminated by meropenem alone. The other two subspaces account for larger pathogen loads. Here, a combination treatment by meropenem and nivolumab is inefficacious for reinvigoration rates below a certain threshold, whereas for larger reinvigoration rates the benefit is mostly moderate. For all nivolumab doses, we see a sharp drop in fitness when pathogen load, *P*_0_, is above 3 × 10^3^ CFU/μL, and raising the dose can only marginally raise this threshold (Figure 6B). As mentioned above, we also see that with *P*_0_ below 3 × 10^3^ CFU/μL, and at a nivolumab dose of 0 mg/kg, treatment benefit is high, confirming that the lower initial pathogen loads allow for successful treatment by antibiotics alone.

### Multiple Dosing

**Figure 7:**
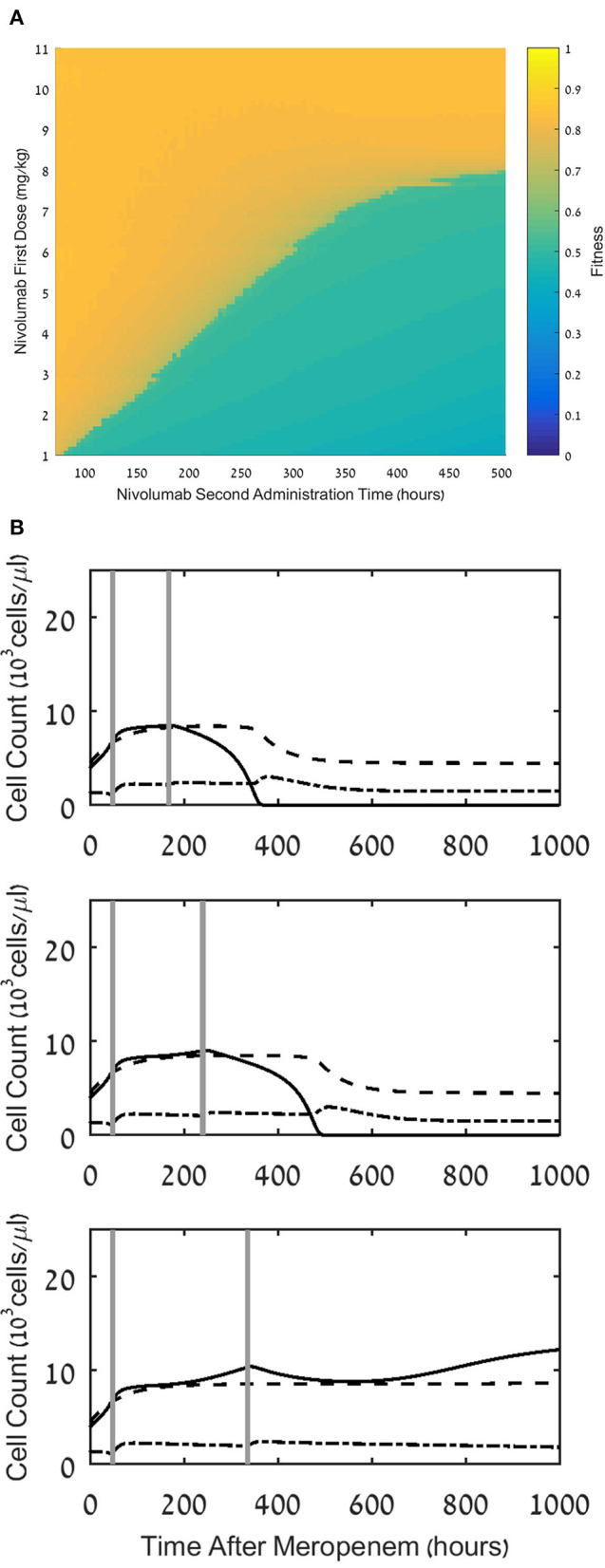
Effect of multiple dosing. **(A)** Fitness values, F, for treatment regimens comprising a first dosing at 48h with a dose, *D*_1_, ranging between 1 and 11 mg/kg, and a second dosing at time ranging between 96 and 504h, with a dose of 12 mg/kg - *D*_1_. **(B)** Model simulations of the three populations of interest: neutrophils (dashed lines), lymphocytes (dash-dot) and pathogen(continuous), under continuous treatment with meropenem (antibiotics), 70 mg/L, i.v. application, administered at t=0, and nivolumab, 12 mg/kg, administered in two doses of 6 mg/kg, with administration times as follows: 24h and 168h (top), 48h and 240h (middle), 24h and 336h (bottom). For equations see Methods section. For parameter values, see legend to Figure 3A and Table 1.

Application of nivolumab, 3 mg/kg, every 2 weeks is the recommended regimen across different cancer indications (28). This administration schedule increased survival of cancer patients, as shown in several phase II/III studies (60). We wished to study the effects of such a strategy on treatment benefit in patients with sepsis. To do this, we first compared a large number of schedules, in which the total dose of 12 mg/kg is fractionated into two doses, first dose applied at 48 h, and second dose applied at various intervals after that, ranging from 2 days to 3 weeks. In Figure 7A, we see the results of these experiments: the small range of fitness values achieved under the various fractionated regimens (0.6 < *F*_*w*_(*S*) < 0.9) indicates that when fixing the first administration time at 48 h, splitting the dose makes little difference, with a small but clear advantage to applying the full 12 mg/kg as early as possible. However, comparing Figure 7A to Figure 4A, one notes that the fractionated regimens have superior fitness to those obtained under a single 12 mg/kg dose at 96 h. This result accentuates the advantage of an early application of ICB, even at a dose which is too low to eradicate the pathogen, but can be complemented by a second dosing within days.

**(A)** Fitness values, *F*, for treatment regimens comprising a first dosing at 48 h with a dose, *D*_1_, ranging between 1 and 11 mg/kg, and a second dosing at time ranging between 96 and 504 h, with a dose of 12 mg/kg-*D*_1_. **(B)** Model simulations of the three populations of interest: neutrophils (dashed lines), lymphocytes (dash-dot) and pathogen (continuous), under continuous treatment with meropenem (antibiotics), 70 mg/L, i.v. application, administered at *t* = 0, and nivolumab, 12 mg/kg, administered in two doses of 6 mg/kg, with administration times as follows: 24 h and 168 h (top), 48 h and 240 h (middle), 24 h and 336 h (bottom). For equations see Methods section. For parameter values, see legend to Figure 3A and Table 1.

This is illustrated in Figure 7B, where we simulated three such regimens, showing that after administering a first dose of 6 mg/kg at 48 h, a second similar dose at 168 h, or 240 h, completes the pathogen elimination. Comparison of this result to Figure 3, middle row, where 6 mg/kg at 48 h by itself fails to eradicate the pathogen, suggests that a second application of nivolumab within 10 days following the first application can do so, albeit after the patient endures a long period of infection, hence the somewhat reduced fitness of this regimen (see Figure 7A).

## Discussion

Sepsis results from a failure of the adaptive immune arm in the race between two antagonistic forces. One force–the pathogen–grows in numbers and progressively suppresses the immune control of its population growth, by exhausting effector CTLs. The opposed force–the adaptive immune arm–struggles to extinguish the pathogen before the latter depletes the effector CTL compartment, proliferates with no constraints and establishes life-risking sepsis (16, 40, 61).

In this work, we evaluated the benefit of a sepsis treatment by the antibiotic drug meropenem, combined with the ICB drug nivolumab, for patients who vary in personal parameters of adaptive immunity, or in initial pathogen load. We evaluated treatment benefit in terms of overall pathogen load, time to pathogen elimination and severity of immunosuppression.

Our results suggest that, within a certain range of initial pathogen load, combined regimens of meropenem and a single application of nivolumab, 6 mg/kg, or more, can succeed in eliminating aggressive bacteria, which would not abdicate to meropenem alone. Such regimens have maximum benefit if the interval between meropenem and nivolumab applications does not exceed a certain dose-dependent threshold; beyond this threshold, the treatment benefit drops sharply. Early nivolumab application is essential for prompt reinvigoration of exhausted CTLs, keeping the depletion of effector CTLs at bay. The nivolumab-reinforced adaptive immune arm eliminates the bacteria efficaciously and returns the system to homeostasis, so that the risk of further serious pathogenesis is minimal. The threshold effect divides the optimization landscapes into two main subspaces, each representing regimens that render maximum or minimum benefit.

The importance of early administration is demonstrated by our analysis of multiple-dose regimens. Within the range of fractionated regimens, all having the same total dose and the same timing of the first nivolumab administration, the fitness function evaluates split dose regimens as less beneficial than a single one, applied at 48 h after meropenem, but superior to a single dose administered at a later time point. This has significant practical implications for ICB therapy protocols. In a scenario where a physician is unsure if the treatment will benefit a certain patient, the strategy of beginning therapy with a smaller dose immediately, and supplementing it later, is preferable to the strategy of waiting longer and applying a large single dose. This conclusion is reinforced by comparing a single application of nivolumab, 6 mg/kg, at 48 h after meropenem—which fails to extinguish the pathogen as monotherapy—to a regimen including a second dosing of nivolumab, 6 mg/kg, within 10 days after the first. Such a regimen succeeds in eliminating the pathogen. Therefore, in cases where damage by the ICB to the patient is conceivable, the physician may prefer a combined meropenem-nivolumab regimen, including two dosing of nivolumab, 6 mg/kg, with up to 10 days interval between them.

Our results further indicate that when initial pathogen load is low (i.e., below 3 × 10^3^ CFU/μL in the present example), antibiotics alone are sufficient for quick pathogen elimination, and the ICB drug is superfluous to need. In contrast, for more severe infections (pathogen load being above 5 × 10^3^ CFU/μL in the present example), the ICB does little to aid the patient's recovery in most cases and other therapeutic strategies should be pursued. The benefit of combined antibiotics and ICB treatment is, therefore, chiefly noticeable within the intermediate range of 3–5 × 10^3^ CFU/μL, or higher, for patients with a relatively high CTL reinvigoration rate (see below). Naturally, the optimal interval between meropenem administration and nivolumab administration is closely associated with the pathogen load, since if the antibiotics fail to eliminate the pathogen, the longer the nivolumab dose is delayed the more the pathogen's population size increases in the interim, and the ICB-boosted immune response will meet it at a higher power.

Agur and colleagues have introduced the theory, and suggested the methodology, for heuristically optimizing the efficacy/toxicity ratio of specific drugs, by integrating dynamic mathematical modeling of drug/host/disease interactions with Operation Research methodology (34). Agur and colleagues applied this optimization methodology to various chemotherapy drugs, measuring efficacy by changes in tumor load, and toxicity, by the most significant related toxicity, essentially, the drug-induced disruption of hematopoiesis [see e.g., (62)]. In the current study, we simulated treatment regimens which included one nivolumab dose, 6 or 12 mg/kg, or two variably fractionated doses, totaling 12 mg/kg, within a 7 days' interval. This dose range is comparable with the nivolumab application, 480 mg or 960 mg, reported for a Phase Ib clinical trial for evaluating safety, tolerability and PK/PD of nivolumab in ICU-admitted patients with sepsis. Most unexpected adverse events found in this clinical trial were mild to moderate, and none were due to nivolumab administration. Cytokine analysis showed no evidence for cytokine storm (19). These findings were corroborated in another clinical trial–a multicenter, open label, phase I/II study (54). It appears, then, that in sepsis, administration of nivolumab in combination with the Standard of Care treatment has not yielded safety findings that justify a compromise of treatment efficacy for the sake of alleviating a safety concern. Accordingly, we chose not to include in the optimization problem the risk of hyper-inflammation, due to the clinical scarcity of cytokine storm under the studied nivolumab regimens. However, we did consider the disease-related adverse effects, e.g., the risk of nosocomial infections, in terms of immunosuppression, aiming to minimize the overall reduction in the number of CTLs below their level in homeostasis. We measured efficacy by drug effect on pathogen load, aiming at both minimizing the overall load and, in addition, minimizing the time to pathogen elimination. Clearly, the exact formulation of the optimization problem, and its associated fitness function, is flexible, and can be determined *ad hoc*, once new adverse effects are discovered, new treatment modalities are introduced, or new treatment aims are set.

The optimal combination regimens, put forward in this work must be examined in prospective clinical trials in ICU-admitted patients with sepsis, harboring persistent, high load, bacteremia, ineffectively treated by Standard of Care antibiotics. However, the fine-tuning of the optimal nivolumab regimen depends on personal parameters, such as viral load at admission, CTL reinvigoration rate, etc. The success of regimen personalization depends on the practicality of evaluating these personal parameters within the medical realm.

Our mathematical model is flexible, pertaining to a wide range of types of antibiotics and ICBs, as well as other therapeutic possibilities, [e.g., (63, 64)]. Broadening the model to include intermediate progenitors and other types of cells, cell signals, and immunophenotypes [e.g., (65, 66)] can improve the assessment of personally measured parameters. In addition, using the model we can also test the feasibility of optimally timing ICB application within a periodic antibiotic treatment. Furthermore, the type of pathogen is also a key element for forecast modeling. Future work will address these issues and will validate these model predictions in animal models as well as in intensive care patients. Of note, in the real world, the reinvigoration process renders exhausted CTLs into functional ones with a distinct acquired epigenetic profile, which depends on the CTL differentiation status, and with an impaired reacquisition of immune memory (67, 68), posing a further challenge on the model. Finally, the aforementioned flexibility of the model means it could feasibly describe the immune response to viral sepsis, e.g., (69–71), with adjustments according to the immune landscape in this form of sepsis, and be used to evaluate applicable therapies for, e.g., SARS-Cov-2 (72).

Precision medicine already has been implicated in bacterial sepsis (73), and it has been suggested that the personal patient parameters can serve as biomarkers for response to the ICB drug, and for determining its necessary application dose (21). In this work, we showed theoretically that personal cellular immunity parameters, notably, those pertaining to CTL reinvigoration, could determine the efficacy of immunotherapy, as well as the merits of increasing the dose of nivolumab, specifically. Our results suggest that patients with a high reinvigoration rate can obtain far better response to ICB, even with initial pathogen load as high as 10 × 10^3^ CFU/μL. Moreover, we show that the larger the reinvigoration rate, the less significant the administration time for a given dose. This suggests that patients with a larger reinvigoration rate would be less susceptible to a delay in the ICB administration, or to its exact dose.

Nivolumab has been developed for oncotherapy, where robust predictive biomarkers for response classification are still lacking (20). Huang et al., (74), suggest that the ratio of the reinvigorated exhausted CTLs to the basic tumor load, positively correlates with clinical response of patients with melanoma to pembrolizumab—another PD-1 blocker—and can serve as a response predictor for this drug. We examined the suitability of this potential combined response marker in sepsis. Our results vary from those of Huang et al. (74), in indicating that even though the reinvigoration rate and the initial pathogen load, independently, affect the quality of response of patients with sepsis to nivolumab, the ratio between these two parameters plays little role in determining the treatment benefit. We inferred this conclusion from Figure 6A, where below a certain initial pathogen load the combined treatment will be equally efficacious for a large range of reinvigoration rates, and above a certain initial pathogen load, treatment is not efficacious, or suboptimal, over large ranges of reinvigoration rates. Only within a narrow intermediate range of initial pathogen loads and reinvigoration rates, a larger initial pathogen load requires a higher reinvigoration rate for extinguishing the pathogen with acceptable efficacy.

In cancer, the parameters controlling the balance between the different forces affecting disease progression can be continuously fine-tuned, by somatic evolution, due to the slow processes characterizing this disease, measured in months or even years. In contrast, in sepsis, measured in a scale of days, short-term abrupt processes with fixed parameters govern this balance. The advantage of this type of control for the therapy of sepsis is the relative ease of liaising concrete parameter values with response to therapy. Specifically, our results suggest that pathogen load, and reinvigoration rate, evaluated at treatment onset, can be two good response predictors and, hence, personalize ICB drug therapy for this condition. We found that other CTL-related parameters play a less important role in determining response. For example, our results indicate that when exhaustion rate is high, large doses of nivolumab increase treatment benefit, and vice versa, low doses evaluated poorly for large rates of exhaustion. However, there is an additive effect of different suppressive mechanisms on functional CTL proliferation, as borne out in Figure 4C. Because the clinical evaluation of different personal suppression mechanisms is not realistic, at present, we suggest evaluating personal response to ICB drugs, solely by the initial pathogen loads and the reinvigoration rate.

The feasibility of using the reinvigoration rate, or the pathogen load, as personal response markers to ICB treatment, should be examined experimentally. The former parameter could be assessed, upon checkpoint blockade, by the expression of Ki-67—a marker of cellular proliferation and T-cell reinvigoration in mouse models and humans (74). How to evaluate the latter parameter is still debated; several suggestions include measurements of infected red blood cells or the plasma concentration of pathogen molecules (bacterial biomass) (75).

It is important to acknowledge the limitations and risks of ICB therapy in sepsis, from both theoretical and practical perspectives. First, these drugs are fundamentally pro-inflammatory, and patients with sepsis are frequently prone to catastrophic organ damage due to major inflammatory events, such as cytokine storms; administering ICBs carries a risk of inducing such an event (76); note, however, that cytokine storm was not observed in the hitherto performed Phase I clinical trials. Furthermore, as reflected in our simulation results, not all patients who avoid excessive inflammation will respond to ICBs sufficiently well to alleviate sepsis. Rather, their response depends on their personal immune attributes and the severity of the infection. In these cases, applying an expensive therapy, such as ICBs, will be unnecessarily wasteful. This, once again, highlights the importance of developing methods for quickly classifying patients according to their expected response to this therapy. In our results, we found various scenarios in which ICB drug therapy is either ineffective or unnecessary. In all those cases, avoiding the risks and costs which come with ICBs would be a significant benefit.

In conclusion, we presented here a new optimization method for assessing the comprehensive value of various combined antibiotics and immunotherapy treatment regimens in alleviating bacterial sepsis. At the core of the method lies a mathematical model for the dynamic interaction of the pathogen with the immune system of the human host, extending the model published by Gillis et al. (33), and including more explicit descriptions of the exhaustion and reinvigoration processes in CTLs, as well as detailed PK/PD of the examined drugs. The findings from applying this optimization method reinforce the importance of early administration of immunotherapy in sepsis, to intercept CTL exhaustion prior to the deterioration of the patient's condition. Specifically, it emerges from our work that a good strategy for alleviating sepsis, if treatment by antibiotics alone is not sufficient, is early administration of one or two doses of nivolumab, 6 mg/kg or 12 mg/kg, within 7–10 days apart, combined with a continuous infusion of antibiotics. Our work underlines the significance of the individual patient's parameters, notably, the pathogen load and the CTL reinvigoration rate, for determining response to treatment by ICB, and suggests that evaluation of those can improve the adjustment of the individual treatment. With our model and optimization method, we hope to provide physicians in intensive care units a valuable tool for identifying the optimal strategy to achieve efficacious sepsis therapy.

## Data Availability Statement

The original contributions generated for this study are included in the article/supplementary material, further inquiries can be directed to the corresponding author/s.

## Author Contributions

AG, AB, and ZA performed the research, wrote and revised the article. All authors contributed to the article and approved the submitted version.

## Conflict of Interest

The authors declare that the research was conducted in the absence of any commercial or financial relationships that could be construed as a potential conflict of interest.

## Footnotes

^1^In the results section the effect of exhausted cells on CTL proliferation is presented as the parameter, $s_{X}^{*}=50•10^{3}$ cells/μ*l* − *s*_*X*_, to have the parameter correlate positively with the suppression, for the sake of clarity.
